# An African visitor in Brazil

**DOI:** 10.1590/S1679-45082013000200022

**Published:** 2013

**Authors:** Jacyr Pasternak, Sergio Barsanti Wey, Paulo Augusto Achucarro Silveira, Thiago Zinsly Sampaio Camargo

**Affiliations:** 1 Hospital Israelita Albert Einstein, São Paulo, SP, Brazil

**Figure 1:**
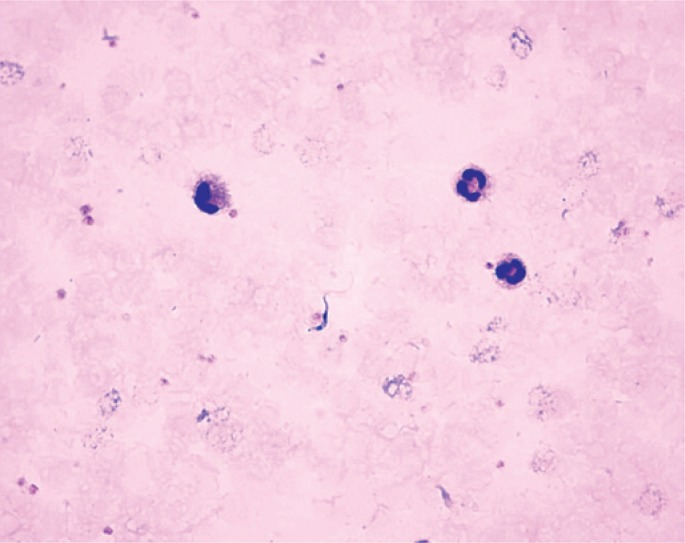
Thick blood smear with trypanosoma

**Figure 2:**
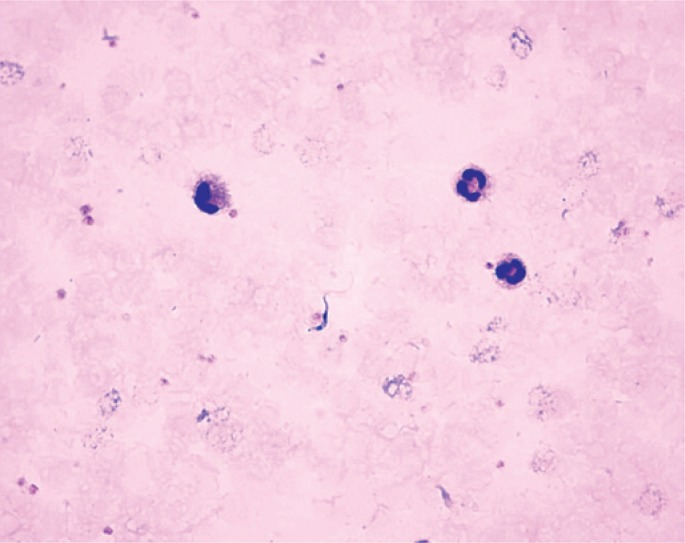
Skin lesion

A Canadian man came to the hospital with history of daily fever in the afternoon, for five days, ranging from 38.5°C to 39°C, headache, muscle pain and asthenia. He is a photographer enthusiastic about wild life and had been to Zimbabwe for 14 days, where he filmed groups of lions and animals of the African megafauna. Upon examination, he presented a hyperemic skin lesion in the posterior region of the thorax, which appeared after an insect bite when he was concluding his visit to Africa, however the lesion persisted until admission to the hospital (Figure 1). A complete blood count and thick blood smear revealed the presence of trypanosomes (Figure 2), probably from the species *Trypanosoma brucei rhodesiense*. The African trypanosomes (*Trypanosoma brucei gambiense* and *Trypanosoma brucei rhodesiense*) are morphologically indistinguishable. The acute disease with the clinical characteristics described above, and the location where the patient was contaminated point to *Trypanosoma rhodesiense*; moreover, *Trypanosoma gambiense* is not easily found in peripheral blood. The African trypanosomes differ from *Trypanosoma cruzi* for having larger and anterior kinetoplasts.

Upon diagnosis, treatment was initiated with pentamidine, with fast response. Later therapy would be completed with suramin to avoid neurological disease. The patient felt better and decided to return to his country, but the Canadian physicians chose to not prescribe suramin, considering early diagnosis and treatment. In the last contact made with the patient six months ago, he was in good conditions and asymptomatic.

There have been some cases of sleeping sickness in visitants of the national parks of Tanzania^(1)^ and Kenya^(2,3)^; and there are some reviews of non-endemic cases^(4)^. Early diagnosis is important, since it is a fatal disease if not treated. If there is central system involvement, patients should be treated with melarprosol, an arsenic-based drug that is highly toxic^(5)^. The disease may be very severe and similar to malaria caused by *Plasmodium falciparum* ^(6)^.
